# Development of a multicentre cohort study to understand the role of MRI and ultrasound in the diagnosis of acute haematogenous bone and joint infection in children (the PIC Bone study): a study protocol

**DOI:** 10.1302/2633-1462.66.BJO-2024-0277

**Published:** 2025-06-10

**Authors:** Marie-Caroline Nogaro, Stuart Hartshorn, Mariea Brady, Amaka Offiah, Saul Faust, Gregory Firth, Jie Ma, Paula Dhiman, Joanna O'Mahoney, Loretta Davies, Catherine Spowart, Amy Moscrop, Bridget Young, Catrin Tudur-Smith, Gary Collins, Daniel C. Perry, Tim Theologis

**Affiliations:** 1 Nuffield Department of Orthopaedics, Rheumatology and Musculoskeletal Sciences, University of Oxford, Oxford, UK; 2 Birmingham Clinical Trials Unit, University of Birmingham, Birmingham, UK; 3 Oxford University Hospitals NHS Foundation Trust, Oxford; 4 Sheffield Children’s NHS Foundation Trust, Sheffield, UK; 5 National Institute of Health Research Southampton Clinical Research Facility and Biomedical Research Centre, University Hospital Southampton NHS Foundation Trust and the University of Southampton, Southampton, UK; 6 Maidstone and Tunbridge Wells NHS Trust, Trauma and Orthopaedics Tunbridge Wells, Kent, UK; 7 University of Liverpool Faculty of Health and Life Sciences, Liverpool, UK; 8 Patient and Public Involvement (PPI), Based in England; 9 Oxford University Hospitals NHS Foundation Trust, Oxford

**Keywords:** Bone and joint infection, Ultrasound scan, Magnetic resonance imaging, Children, Osteomyelitis, Septic arthritis, joint infections, MRI, cohort study, ultrasound scans, Diagnostic test, ultrasound in the diagnosis, logistic regression, clinicians, bone infections, orthopaedic surgery

## Abstract

**Aims:**

Bone and joint infections (BJI) in children are rare but can be serious. Differentiating BJI from other conditions with similar symptoms is critical. Advanced imaging (ultrasound scans (USS) and MRI) is often required to confirm the diagnosis. The differing merits of imaging type and regional variation in access to advanced imaging can lead to diagnostic uncertainty and treatment variation. The aim of this study is to evaluate the diagnostic accuracy of MRI and USS for the investigation of BJI in children, and develop and validate prediction models to aid the diagnosis of BJI in children. A nested qualitative sub-study will explore acceptability of the imaging to children, parents, and health practitioners.

**Methods:**

A multicentre retrospective cohort of children (aged < 16 years) with suspected diagnosis of BJI will be used to estimate the diagnostic accuracy of the two imaging methods and develop the prediction models. The models will be evaluated in a second cohort of prospectively recruited children. Diagnostic test accuracy will be estimated overall, and separately for children aged under and over five years. The prediction models will be fit using logistic regression, with candidate predictors chosen based on clinical plausibility and from a review of the literature. Continuous predictors will be examined for non-linearity with confirmed BJI using fractional polynomials. Multiple imputation will be used to replace missing values. Internal validation will be carried out using bootstrapping. Model performance will be assessed with discrimination and calibration.

**Discussion:**

Ethical approval for this study (registration: ISRCTN15471635) was granted (REC reference 23/WM/0027). Informed consent is being obtained from participants in the prospective cohort and the qualitative sub-study. Study findings will be published in an open access journal and presented at relevant national and international conferences. Relevant charities and associations are being engaged to promote awareness of the project.

Cite this article: *Bone Jt Open* 2025;6(6):677–684.

## Introduction

Osteomyelitis is a bacterial infection of bone. In children, it is typically introduced through the blood (haematogenous). Acute osteomyelitis presents with symptoms of less than two weeks’ duration, including pain, loss of limb function, raised temperature, and malaise. The infection frequently ‘breaks out’ into the adjacent joint, causing ‘septic arthritis’, although joint sepsis can also be haematogenous.^1^ Osteomyelitis and septic arthritis in children are inextricably linked and considered together as ‘osteoarticular infection’ or bone and/or joint infection. The burden of bone and joint infection is significant, with data suggesting that approximately 1,800 children (aged 0 to 16 years) are admitted to hospitals in England each year.^2^ Untreated bone and joint infection rapidly progresses to irreversible joint cartilage and/or growth plate damage and bone destruction, leading to a limb-threatening situation, while systemic sepsis can have life-threatening implications. Early differentiation of bone and joint infection from less urgent conditions mimicking the symptoms, such as transient synovitis, is critical.

Clinical work-up typically includes the history of the illness, clinical examination, routine blood tests, and radiographs. However, despite this work-up, clinicians are frequently left facing difficulties in distinguishing bone and joint infection from the other potential causes, and therefore consider advanced imaging. Such imaging includes ultrasound scan (USS) and MRI. USS is more readily accessible and can identify organized fluid collections within joints or tissues, but the overall diagnostic value is unknown. MRI is the gold-standard investigation to diagnose bone and joint infection in adults,^3^ but its diagnostic value is unknown in children. Furthermore, MRI often requires sedation or anaesthesia in children, which influences clinical decision-making.

A clear pathway outlining which tests to perform, and when they are needed, would help to ensure that bone infections are not missed. This would also reduce unnecessary tests in children who do not have an infection and get to a diagnosis more quickly. A recent comprehensive review, through a Health Technology Assessment, concerning advanced imaging for bone and joint infection did not identify any studies of relevance to children.^3^ The Paediatric Emergency Medicine Collaborative in the UK and Ireland (PERUKI)^4,5^ and the Australian Paediatric Emergency Medicine Research Collaborative^6^ have prioritized improving decision support tools, to help differentiate bone and joint infection from other diagnoses, among their top ten most important research areas. In particular, better guidance regarding the use of imaging to optimize the diagnosis of bone and joint infection in children would be valued by clinicians and families.^6-8^

The aim of this study is to evaluate the diagnostic accuracy of MRI and ultrasound scans for the investigation of bone and joint infection in children, and to develop and validate two prediction models (one for children aged under five years, and one for children aged five years and older) to aid the diagnosis of bone and joint infection in children. The prediction models will be developed on the basis of retrospective data of children investigated for infection. They will then be validated on a prospective cohort of children undergoing investigations for bone and joint infection.

## Methods and analysis

### Design

The study will consist of two phases:

1. A multicentre retrospective cohort study based on hospital records to establish the diagnostic accuracy of MRI and USS, and to develop a clinical prediction model to better inform the diagnosis of bone and joint infection; and
2. A multicentre prospective cohort study to externally validate the clinical prediction model.

In parallel, a qualitative study will aim to inform the management of patients being investigated for bone and joint infection osteomyelitis, including how best to address their information needs and how to support them during the diagnostic process.

The selection criteria and data collection are nearly identical in both the retrospective and prospective cohorts. We have established the feasibility of reliable retrospective data collection through a pilot study.

### Study population

The target population is children and young people aged under 15 years and nine months (aged under 16 years at the end of the three-month follow-up) with a suspected diagnosis of bone and joint infection by the treating clinician. The cohort study will be conducted in NHS secondary care hospitals throughout the UK. The first cohort (retrospective) will be identified through emergency department (ED) records in participating centres. The second cohort (prospective) will also be recruited in ED or acute wards at the same centres. Support from PERUKI and the British Society for Children’s Orthopaedic Surgery (BSCOS)^9^ will help facilitate recruitment and support the study.

We have considered the Innovations in Clinical Trial Design and Delivery for the Under-served (INCLUDE) guidance in preparing the study.^10^ Diversity is particularly important, as diseases which appear more frequently in different racial or ethnic groups may affect the diagnostic process of bone and joint infection (e.g. sickle cell disease, haemophilia). Furthermore, there is evidence that the incidence of bone and joint infection increases with increasing socioeconomic deprivation.^11^ In discussion with our Patient and Public Involvement co-investigators, we will be targeting hospitals covering under-served and ethnically and racially diverse areas (e.g. East London, Birmingham) to ensure our sample is inclusive.

The main inclusion and exclusion criteria for the trial are summarized in Table I.

**Table I.** Inclusion and exclusion criteria.

Inclusion criteria	Exclusion criteria
Age 0 to 15 years	There is evidence that the patient and/or parent/guardian would be unable to adhere to study procedures or complete follow-up
Bone and joint infection is part of the differential diagnosis, even if the treating clinician believes it can be ruled out on the basis of history and examination alone	Limited comprehension by the parent/guardian of the English language
Duration of symptoms < two weeks at the time of attendance to acute healthcare	Suspected infections affecting the axial skeleton (skull, spine, or ribs)
Symptoms affecting the appendicular skeleton only	Traumatic aetiology of symptoms

We have established the feasibility of reliable retrospective data collection through a pilot study.

### Reference standard for diagnosis of bone and joint infection

This is based on a previous large National Institute for Health and Care Research (NIHR)-funded cohort study (DINOSAUR)^7^ and a NIHR-funded Health Technology Assessment of osteomyelitis imaging.^3^ However, several scenarios exist where the prior published reports may not fully inform the reference standard in the context of the current study. To address these gaps, the study management group predefined additional rules, informed by literature, notably:

- Conventional radiographs are initially normal in more than 50% of children with osteomyelitis,^7^ although radiological changes typically become apparent within seven to ten days of bacterial joint infection.^12^
- Spontaneous resolution of bone and joint infection is exceedingly rare, meaning that symptom resolution without antibiotic therapy or surgical intervention would effectively rule out bone and joint infection.^13^

Based on these considerations, the definitive rules for defining bone and joint infection were:

Definitive diagnosis of bone and joint infection, including:

- Positive polymerase chain reaction (PCR) joint aspirate; and/or
- Positive histopathology or microbiology on bone/joint specimen; and/or
- Positive blood culture with no other source of infection; and/or
- Joint aspirate > 50,000 white blood cells (WBCs); and/or
- Evidence of bone and joint infection on two imaging methods or on follow-up imaging.

Definitive exclusion of bone and joint infection, including:

- Clinical resolution of symptoms without antibiotic treatment by three months. Less than 24 hours of treatment dose antibiotics is considered as ‘no’ antibiotic use; and/or
- Confirmation of an alternative diagnosis to explain symptoms subsequently established.

Uncertain group, including:

- Children with negative or equivocal diagnostic investigations – recovered following more than 24 hours of antibiotic treatment.

### Screening, eligibility assessment, and recruitment

Retrospective cohort for model development: Site ED records will be screened for potential cases of bone and joint infection by the local clinical team. ED attendance codes/keywords including, but not limited to, any of the keywords ‘limp, limping child, abnormal gait, osteomyelitis, septic arthritis, swollen leg/joint, non-weightbearing, limb and joint pain, transient synovitis’ will be reviewed. These predefined attendance codes and keywords were derived during the feasibility study, but additional keywords can be identified and added by the participating centres. If the ED record indicates bone and joint infection as a differential diagnosis, the electronic patient record will be reviewed. Patient medical records, which include their admission and progress notes, operation notes, discharge summaries, and imaging reports will be scrutinized.

**Fig. 1:**
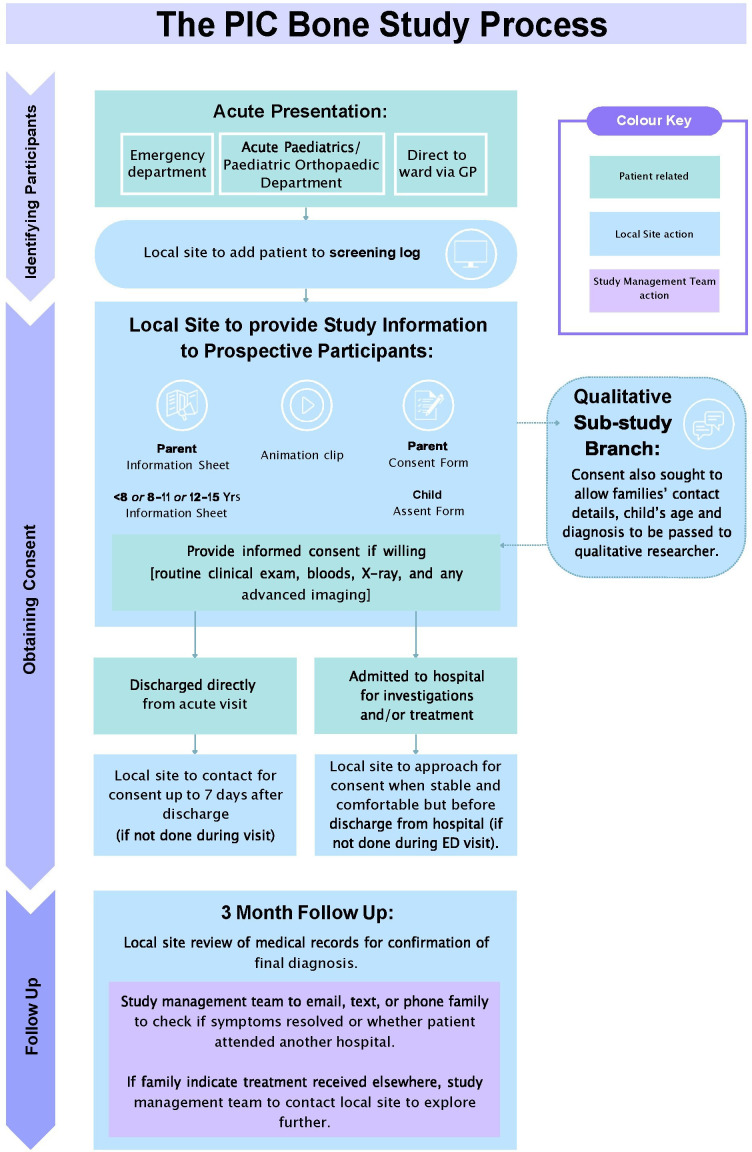
Patient flowchart (prospective cohort).

Prospective cohort (18 months recruitment – three months’ follow-up) for model validation: Similar to the retrospective cohort, patients with suspected bone and joint infection will be identified in participating centres either in the ED, on admission, or in the paediatric or paediatric orthopaedic department. Patients will be recruited when the treating clinician believes that the child may have a diagnosis of bone and/or joint infection (i.e. bone and joint infection is part of the differential diagnosis, even if the treating clinician believes it can be effectively ruled out based on the history and examination alone). At three months post recruitment, information pertinent to defining the reference standard will be collected. Any complications will be recorded. The DINOSAUR study demonstrated that the diagnosis of osteomyelitis can be reliably determined by three months.^7^ Families of children discharged from hospital without a definitive diagnosis will be contacted by text message, phone, or email at three months from recruitment to confirm complete resolution of symptoms and ensure they were not treated in a different hospital. The patient flowchart (prospective cohort only) is shown in Figure 1.

Qualitative study: The qualitative study will run in approximately 15 of the sites involved in the prospective cohort study and explore the perspectives of children/young people, parents/carers, and health professionals on the diagnostic process for bone and joint infection. Participants from the prospective cohort will be invited to participate in this sub-study.

We will conduct semi-structured qualitative interviews with a purposive sample of approximately 20 families, sampling for diversity in terms of study site, child age, ethnicity, and family socioeconomic status, and include patients with experience of USS, MRI, or both. The sample will mainly comprise those diagnosed with bone and joint infection, but we will also interview a sub-set of patients/families who have undergone investigations for suspected bone and joint infection but received other diagnoses. The perspectives of these two groups may differ in important ways, and it is important that clinical practice is informed by both groups. Sampling of families will also be informed by the concept of information power,^11^ and the ongoing data analysis.

### Sample size considerations

Sample size for retrospective cohort to estimate the diagnostic accuracy: For the primary objective, we will collect retrospective data for an estimated sample of 6,000 children with suspected bone and joint infection. Based on our feasibility data, approximately 444 (7.4%) would have confirmed bone and joint infection and 5,556 (92.6%) would be disease-free. Based on our pilot study, this would provide data for approximately 351 (79%) MRI disease cases and 408 (92%) USS disease cases, allowing MRI sensitivity to be estimated to within 2.9 percentage points of 91.5% and USS sensitivity to be estimated to within 4.3 percentage points of 74.1% with 95% confidence. This would also provide 611 (11%) MRI and 1,000 (18%) USS disease-free cases, allowing specificity to be estimated to within 3.7 percentage points of 67% for MRI and within 3.1 percentage points of 47% for USS with 95% CI.

Sample size for retrospective cohort to develop the diagnostic prediction model: We will develop two diagnostic prediction models to predict bone and joint infection; one for children aged under five years (Model 1) and one for children aged five years and older (Model 2).

In the retrospective cohort, we anticipate that there will be 1,440 children aged under five years, of whom 107 have a bone and joint infection for Model 1, and 4,560 children aged five years and older, of whom 348 have a bone and joint infection for Model 2. This is based on the estimate that 24% of children with confirmed bone and joint infection will be aged under five years (NHS Digital 2017/18).^14^

Using this information and to minimize the risk of overfitting and to precisely estimate the overall outcome risk, we calculated that ten predictors can be examined for inclusion in Model 1 and 32 predictors can be examined for inclusion in Model 2. This is based on a conservative estimate of the anticipated R^2^ of 0.15*max(R^2^) (max R^2^ = 0.41 for an anticipated outcome prevalence of 0.074).^15^

We have since updated the sample size calculation during data collection based on changes in the prevalence of bone and joint infection from 7.4% to 5.3% for Model 1 and to 7.6% for Model 2 (change made 9 October 2024). With the current record of the retrospection cohort, there are 3,426 children aged under five years, of whom 180 have bone and joint infection (Model 1), and 2,314 children aged five years and older, of whom 177 have bone and joint infection (Model 2). Based on a conservative estimate of the anticipated R^2^ of 0.15*max(R^2^) (max R^2^ = 0.41 for an anticipated outcome prevalence of 0.053 and 0.076), 19 predictors can be examined for inclusion in Model 1 and 16 predictors can be examined for inclusion in Model 2.

Sample size for prospective cohort to evaluate the performance of the diagnostic prediction model: For an external validation study, it was recommended that there are at least 100 events (i.e. instances of bone and joint infection) to evaluate the discrimination and the calibration of the developed model in an independent sample.^16^ The DINOSAUR study recruited at least one child admitted with suspected bone and joint infection from each centre every month.^7^ We therefore plan to recruit 111 patients from at least 30 centres over 18 months to meet the required sample size.

However, since planning this study, new sample size guidance for external validation studies has become available.^17^ Using this new guidance and the updated prevalence estimates for bone and joint infection, we base our sample size calculation using the anticipated c-statistic by the max Nagelkerke’s R^2^ and assume a 5.3% and 7.6% of event rates for Model 1 and Model 2, respectively (change made 20 November 2024). We use 15% of the max R^2^ which equates to ~ 0.05 (Model 1) and 0.06 (Model 2) given the assumed outcome prevalences, and we base our sample size on estimating a c-statistic of ~ 0.77 (Model 1) and 0.75 (Model 2) with targeted a standard error of 0.038. This corresponds to a CI of width 0.15, meaning we would need a sample size of 751 children aged under five years, of whom 40 have a bone and joint infection (Model 1), and 575 children aged five years and older, of whom 44 have a bone and joint infection (Model 2) for external validation.

### Statistical analysis

Diagnostic test accuracy: For each index test (USS and MRI), the results will be compared to the reference standard among all patients with available data and cross tabulations reported (including indeterminate results). The sensitivity, specificity, negative predictive value, positive predictive value, likelihood ratio, and diagnostic odds ratios will be reported (with 95% CIs) overall, as well as separately for patients aged five years and older versus those aged under five years. This choice was based on our feasibility study finding that most children under the age of five years require sedation or anaesthesia for MRI, while those aged five years and above do not.

Model development and validation: Two models will be fitted using logistic regression: one for children aged under five years and one for children aged five years and older. A single unified model will also be explored. Candidate predictors for both models will be chosen based on clinical plausibility and from a review of the literature. We will select variables for inclusion in the model using least angle selection and shrinkage operator (LASSO) penalties. Continuous predictors will be examined for nonlinear relationship with confirmed bone and joint infection using fractional polynomials.^18^ Missing data will be inevitable with not all patients providing data on all predictors of interest.

To avoid excluding patients when developing and validating our models, we will use multiple imputation to impute missing values, under a missing at random assumption. Identifying the true underlying missing data mechanism from the available data is rarely possible. Assumptions need to be made on the plausible mechanism, and approaches needed to be used. Under a missing at random (MAR) assumption, the missingness after conditioning on the observed data does not depend on the unobserved (unseen) data. Using this approach, we can apply methods such as multiple imputation, by fitting a joint model to the observed data and impute the missing data, taking account of the uncertainty in the estimated parameters of this joint model. MAR is a pragmatic decision, which makes a less strong and more realistic assumptions than a missing completely at random approach and conducting a complete-case analysis. The MAR imputation model will include all variables considered for the multivariable model building, the outcome, and any auxiliary variables (such as centre-specific covariates) that will help explain the missingness.

The internal validity of the final models will also be assessed using bootstrap resampling to adjust for over-optimism in the estimation of model performance.^19^ The internal validation will quantify and be used to adjust the performance measures for any optimism. The performance of the prediction model will be characterized by assessing calibration and discrimination.^20^ Calibration, which reflects how close the predictions from the model are to the observed outcome frequencies, will be assessed graphically, using a calibration plot, plotting observed outcomes against predictions using smoothing techniques. The plot will also be supplemented with results for individuals grouped by similar probabilities (tenths) comparing the mean predicted probability to the mean observed outcome. Calibration will also be quantified by calculating the calibration slope and intercept. The discrimination of the prediction models will be summarized with the concordance index (equivalent to the area under receiver operating characteristic curve) with 95% CI.

External validation: The performance of the two models will also be assessed in the prospective external validation cohort.^21^ Discrimination, calibration, and a decision curve analysis will be evaluated. Any miscalibration identified during this phase will be addressed by recalibrating the model (e.g. re-estimating the intercept or updating the regression coefficients by a common factor).^22^ During the external validation the incremental value of adding USS and MRI to both models will be examined. Missing data will be handled using multiple imputation and model performance measures (concordance index, calibration slope, and intercept) will be pooled using Rubin’s rules.

All statistical analyses will be carried out in R v. 4.4.2 (R Project for Statistical Computing, Austria).

## Ethics

The study was approved by Solihull Research Ethics Committee on 28 March 2023 (REC reference 23/WM/0027) and Health Research Authority approval was granted on 28 March 2023.

The prospective cohort and qualitative study will require the participation of children (aged under 16 years). Consent will be obtained from the parents/legal guardians of the children for participation in the study. Information for parents/guardians will give guidance to ensure that they understand the nature of the study and what would be required of them and their children in terms of follow-up. While consent is not obtained from the children, age-appropriate information sheets will actively involve the children in the study process. We will seek assent from all children whom the clinical team believe is competent to understand the study process, which we anticipate will be most children from eight years old. If the child actively declines participation, their wishes will be respected, and we will not include them in the study.

The retrospective cohort within this project will collect anonymous data only. There is no potential for harm to participants, or incentives to participants. Inclusion of children’s data in the study will not affect post-study access to interventions, care, or benefits. The harmonized arrangement for the governance of research ethics committees (GAfREC) has judged that the use of anonymized information in this way is acceptable without requiring patient consent.^23^ The study will be conducted in accordance with the principles of the Medical Research Council’s Good Clinical Practice guidelines and the Declaration of Helsinki.^24^ There is Clinical Trial Unit (CTU) involvement for this study. The CTU will provide expertise in study management, information systems, data management, monitoring, logistics, biostatistics, and reporting. This will include submission for ethical approval, development and maintenance of the electronic database, setup of sites, collection, and data analysis.

## Dissemination

The findings of the study will inform NHS clinical practice for imaging of children with suspected bone and joint infection. We will disseminate the results across the wider health professionals’ community, National Institute for Health and Care Excellence (NICE), and policy makers, and to the public via parent groups/charities. Results of the diagnostic test accuracy of MRI and USS will be reported following the STARD guideline.^25^ The reporting of the development and validation of the clinical prediction model will follow the TRIPOD + AI reporting guideline.^26^ We aim to disseminate the results of this study to all professional societies relevant to the specialities involved in the care of children with bone and joint infection, which has broad representation across emergency medicine, orthopaedic surgery, paediatrics, and radiology. We will support the patient and public involvement partners to help ensure that the information is broadly accessible and give them opportunities to share their insights about the research findings and research process. This study will produce a clear recommendation for NICE guidelines.

### Implementation

Dissemination is important but implementation of the findings is often challenging. To demonstrate broad generalizability of the findings, we have elected to pursue a study with a large sample and a design which covers all stages of the clinical pathway, with a broad array of clinical subspecialities involved in the investigation and management of bone and joint infection (ED doctors, radiologists, paediatricians, orthopaedic surgeons). We believe that our multidisciplinary study team can assess the whole pathway of bone and joint infection management and deliver convincing evidence for all health professionals to use. Informing general practitioners through their professional networks will also be of major importance in changing practices in this condition.

This is an abridged protocol of the PIC Bone study to highlight the important clinically relevant elements of the study. The full working protocol, including details of iterative changes made throughout the development of the study, is available from the NIHR website.

**Take home message**

- Decision support tools to help clinicians differentiate bone and joint infection from other conditions with similar symptoms are rudimentary and do not consider the role of advanced imaging.

- This study is the first to look at the role, specificity, and sensitivity of advanced imaging in the diagnosis of bone and joint infection in children.

- Improved guidance regarding the use of imaging in the diagnosis of bone and joint infection in children is a priority for both clinicians and families.

- There is significant variation in the choice and timing of advanced imaging in children with suspected bone and joint infection. The development of tools to help non-specialist clinicians at the initial point of contact should improve the speed and accuracy of diagnosis and reduce delays to treatment.

## Data Availability

The data that support the findings for this study are available to other researchers from the corresponding author upon reasonable request.

## References

[1] Manz N Krieg AH Heininger U Ritz N Evaluation of the current use of imaging modalities and pathogen detection in children with acute osteomyelitis and septic arthritis Eur J Pediatr 2018 177 7 1071 1080 10.1007/s00431-018-3157-3 29728840

[2] No authors listed Hospital admitted patient care activity, 2017-18 NHS England https://digital.nhs.uk/data-and-information/publications/statistical/hospital-admitted-patient-care-activity/2017-18 date last accessed 29 May 2025

[3] Llewellyn A Jones-Diette J Kraft J Holton C Harden M Simmonds M Imaging tests for the detection of osteomyelitis: a systematic review Health Technol Assess 2019 23 61 1 128 10.3310/hta23610 31670644 PMC6843114

[4] No authors listed Paediatric Emergency Research in the UK and Ireland (PERUKI) https://www.peruki.org/ date last accessed 29 May 2025

[5] Hartshorn S O’Sullivan R Maconochie IK et al. Establishing the research priorities of paediatric emergency medicine clinicians in the UK and Ireland Emerg Med J 2015 32 11 864 868 10.1136/emermed-2014-204484 25678575

[6] Deane HC Wilson CL Babl FE et al. Predict prioritisation study: establishing the research priorities of paediatric emergency medicine physicians in Australia and New Zealand Emerg Med J 2018 35 1 39 45 10.1136/emermed-2017-206727 28855237

[7] de Graaf H Sukhtankar P Arch B et al. Duration of intravenous antibiotic therapy for children with acute osteomyelitis or septic arthritis: a feasibility study Health Technol Assess 2017 21 48 1 164 10.3310/hta21480 28862129 PMC5592430

[8] Hartshorn S O’Sullivan R Maconochie IK et al. Establishing the research priorities of paediatric emergency medicine clinicians in the UK and Ireland Emerg Med J 2015 32 11 864 868 10.1136/emermed-2014-204484 25678575

[9] No authors listed British Society for Children's Orthopaedic Surgery (BSCOS) https://www.bscos.org.uk/ date last accessed 29 May 2025

[10] No authors listed Improving inclusion of under-served groups in clinical research: guidance from INCLUDE project National Institute for Health and Care Research (NIHR) https://www.nihr.ac.uk/improving-inclusion-under-served-groups-clinical-research-guidance-include-project date last accessed 29 May 2025

[11] Malterud K Siersma VD Guassora AD Sample size in qualitative interview studies: guided by information power Qual Health Res 2016 26 13 1753 1760 10.1177/1049732315617444 26613970

[12] van Schuppen J van Doorn MMAC van Rijn RR Childhood osteomyelitis: imaging characteristics Insights Imaging 2012 3 5 519 533 10.1007/s13244-012-0186-8 22875760 PMC3443272

[13] Yeo A Ramachandran M Acute haematogenous osteomyelitis in children BMJ 2014 348 g66 10.1136/bmj.g66 24446020

[14] No authors listed Data sets: 2017-2018 NHS England https://digital.nhs.uk/data-and-information/data-collections-and-data-sets/data-sets date last accessed 29 May 2025

[15] Riley RD Snell KI Ensor J et al. Minimum sample size for developing a multivariable prediction model: part II - binary and time-to-event outcomes Stat Med 2019 38 7 1276 1296 10.1002/sim.7992 30357870 PMC6519266

[16] Collins GS Ogundimu EO Altman DG Sample size considerations for the external validation of a multivariable prognostic model: a resampling study Stat Med 2016 35 2 214 226 10.1002/sim.6787 26553135 PMC4738418

[17] Riley RD Debray TPA Collins GS et al. Minimum sample size for external validation of a clinical prediction model with a binary outcome Stat Med 2021 40 19 4230 4251 10.1002/sim.9025 34031906

[18] Royston P Model selection for univariable fractional polynomials Stata J 2017 17 3 619 629 29398979 PMC5796635

[19] Collins GS Dhiman P Ma J et al. Evaluation of clinical prediction models (part 1): from development to external validation BMJ 2024 384 e074819 10.1136/bmj-2023-074819 38191193 PMC10772854

[20] Steyerberg EW Vickers AJ Cook NR et al. Assessing the performance of prediction models: a framework for traditional and novel measures Epidemiology 2010 21 1 128 138 10.1097/EDE.0b013e3181c30fb2 20010215 PMC3575184

[21] Riley RD Archer L Snell KIE et al. Evaluation of clinical prediction models (part 2): how to undertake an external validation study BMJ 2024 384 e074820 10.1136/bmj-2023-074820 38224968 PMC10788734

[22] Su TL Jaki T Hickey GL Buchan I Sperrin M A review of statistical updating methods for clinical prediction models Stat Methods Med Res 2018 27 1 185 197 10.1177/0962280215626466 27460537

[23] No authors listed Governance arrangements for research ethics committees Health Research Authority 2023 https://www.hra.nhs.uk/planning-and-improving-research/policies-standards-legislation/governance-arrangement-research-ethics-committees/ date last accessed 30 May 2025

[24] No authors listed Declaration of Helsinki World Medical Association https://www.wma.net/policies-post/wma-declaration-of-helsinki/ date last accessed 15 May 2025 10.1016/j.arcmed.2025.103237

[25] Cohen JF Korevaar DA Altman DG et al. STARD 2015 guidelines for reporting diagnostic accuracy studies: explanation and elaboration BMJ Open 2016 6 11 e012799 10.1136/bmjopen-2016-012799 28137831 PMC5128957

[26] Collins GS Moons KGM Dhiman P et al. TRIPOD+AI statement: updated guidance for reporting clinical prediction models that use regression or machine learning methods BMJ 2024 385 e078378 10.1136/bmj-2023-078378 38626948 PMC11019967

